# Supplementary figures and images for: Assessment of phylo-functional coherence along the bacterial phylogeny and taxonomy
Source: Sci Rep. 2021 Apr 15;11:8299. doi: 10.1038/s41598-021-87909-1 (PMC8050241; doi:10.1038/s41598-021-87909-1)

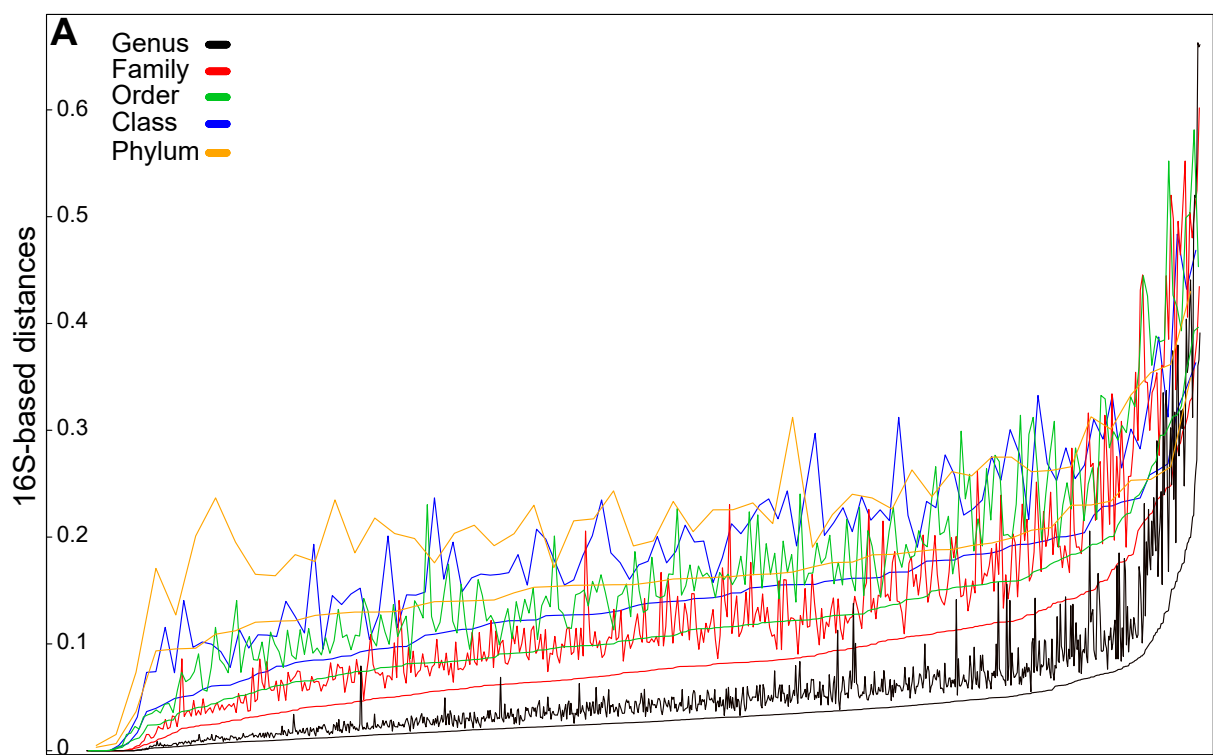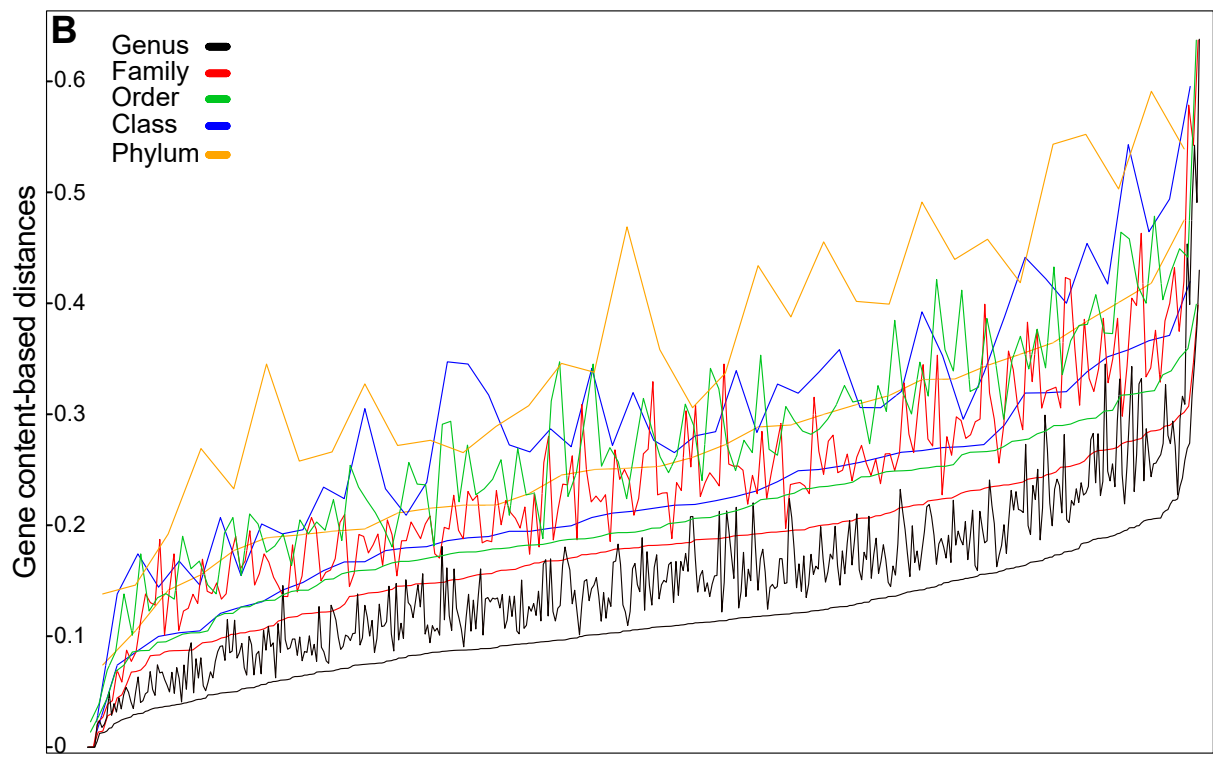

Supplement: Supplementary file 4 — Supplementary Information 4. [file 41598_2021_87909_MOESM4_ESM.pdf]

**Bacteria**  
Node13643

**Actinobacteria**  
Node21110

Node5

Node24782

**Bacteria**  
Node6

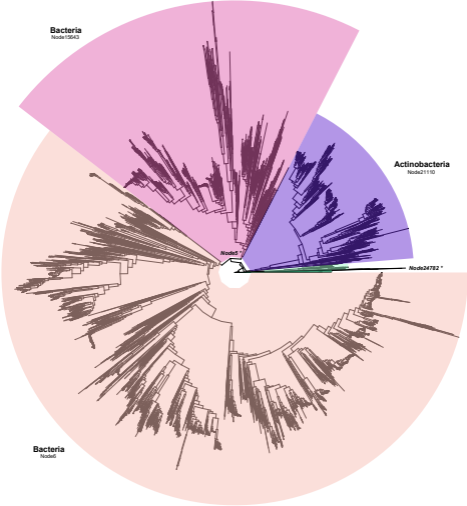

Supplement: Supplementary file 6 — Supplementary Information 6. [file 41598_2021_87909_MOESM6_ESM.pdf]
