# Supplementary material for: Assessment of phylo-functional coherence along the bacterial phylogeny and taxonomy
Source: Sci Rep. 2021 Apr 15;11:8299. doi: 10.1038/s41598-021-87909-1 (PMC8050241; doi:10.1038/s41598-021-87909-1)

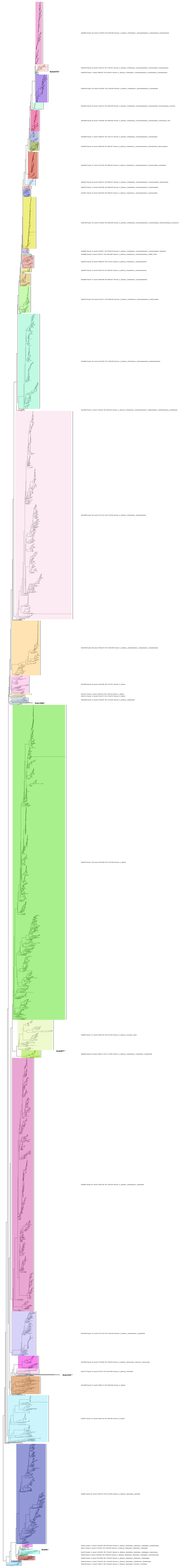

Supplement: Supplementary file 5 — Supplementary Information 5. [file 41598_2021_87909_MOESM5_ESM.pdf]
