# Supplementary material for: Assessment of phylo-functional coherence along the bacterial phylogeny and taxonomy
Source: Sci Rep. 2021 Apr 15;11:8299. doi: 10.1038/s41598-021-87909-1 (PMC8050241; doi:10.1038/s41598-021-87909-1)
Supplement: Supplementary file 7 — Supplementary Information 7. [file 41598_2021_87909_MOESM7_ESM.pdf]

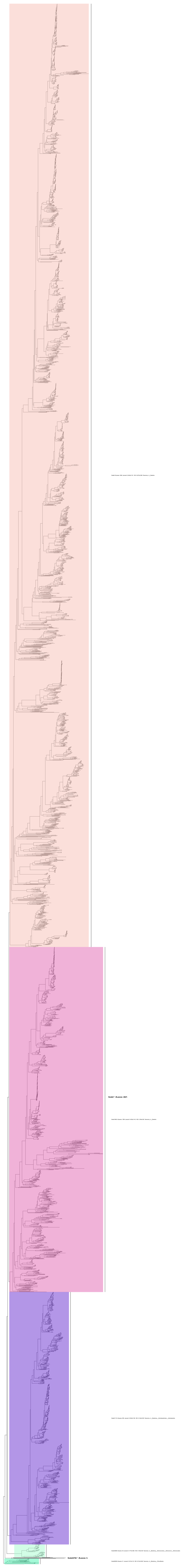

Node6. #Leaves: 3549. Jaccard: 0.454±0.101. 16S: 0.227±0.058. Taxonomy: ;d__Bacteria
Node5 *. #Leaves: 4847.
Node15643. #Leaves: 1298. Jaccard: 0.433±0.143. 16S: 0.184±0.06. Taxonomy: ;d__Bacteria
Node21110. #Leaves: 950. Jaccard: 0.332±0.108. 16S: 0.134±0.053. Taxonomy: ;d__Bacteria;p__Actinobacteriota;c__Actinobacteria
Node24689. #Leaves: 48. Jaccard: 0.177±0.056. 16S: 0.154±0.06. Taxonomy: ;d__Bacteria;p__Deinococcota;c__Deinococci;o__Deinococcales
Node24782 *. #Leaves: 5.
Node26938. #Leaves: 21. Jaccard: 0.331±0.16. 16S: 0.212±0.065. Taxonomy: ;d__Bacteria;p__Chloroflexota
